# Dinosaur Footprints and Other Ichnofauna from the Cretaceous Kem Kem Beds of Morocco

**DOI:** 10.1371/journal.pone.0090751

**Published:** 2014-03-06

**Authors:** Nizar Ibrahim, David J. Varricchio, Paul C. Sereno, Jeff A. Wilson, Didier B. Dutheil, David M. Martill, Lahssen Baidder, Samir Zouhri

**Affiliations:** 1 Department of Organismal Biology and Anatomy, University of Chicago, Chicago, Illinois, United States of America; 2 Department of Earth Sciences, Montana State University, Bozeman, Montana, United States of America; 3 Museum of Paleontology and Department of Earth and Environmental Sciences, University of Michigan, Ann Arbor, Michigan, United States of America; 4 15 Passage du Buisson Saint-Louis, Paris, France; 5 School of Earth and Environmental Sciences, University of Portsmouth, Portsmouth, United Kingdom; 6 Laboratoire de Géosciences, Faculté des Sciences Aïn Chock, Université Hassan II, Casablanca, Morocco; Raymond M. Alf Museum of Paleontology, United States of America

## Abstract

We describe an extensive ichnofossil assemblage from the likely Cenomanian-age ‘lower’ and ‘upper’ units of the ‘Kem Kem beds’ in southeastern Morocco. In the lower unit, trace fossils include narrow vertical burrows in cross-bedded sandstones and borings in dinosaur bone, with the latter identified as the insect ichnotaxon *Cubiculum ornatus*. In the upper unit, several horizons preserve abundant footprints from theropod dinosaurs. Sauropod and ornithischian footprints are much rarer, similar to the record for fossil bone and teeth in the Kem Kem assemblage. The upper unit also preserves a variety of invertebrate traces including *Conichnus* (the resting trace of a sea-anemone), *Scolicia* (a gastropod trace), *Beaconites* (a probable annelid burrow), and subvertical burrows likely created by crabs for residence and detrital feeding on a tidal flat. The ichnofossil assemblage from the Upper Cretaceous Kem Kem beds contributes evidence for a transition from predominantly terrestrial to marine deposition. Body fossil and ichnofossil records together provide a detailed view of faunal diversity and local conditions within a fluvial and deltaic depositional setting on the northwestern coast of Africa toward the end of the Cretaceous.

## Introduction

**Figure 1:**
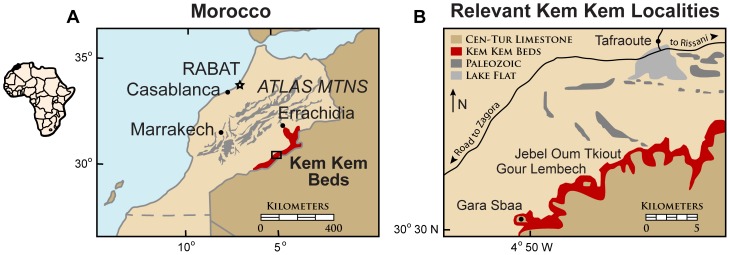
Geographical location of the Kem Kem ichnofossils. A, Location of Morocco (left corner of Africa map) and the Kem Kem outcrops shown in red, modified from Sereno and Larsson (2009) [56]. B, Close-up of Kem Kem localities located near the main ichnofossil site, Gara Sbaa (area shown as rectangle in A.)

**Figure 2:**
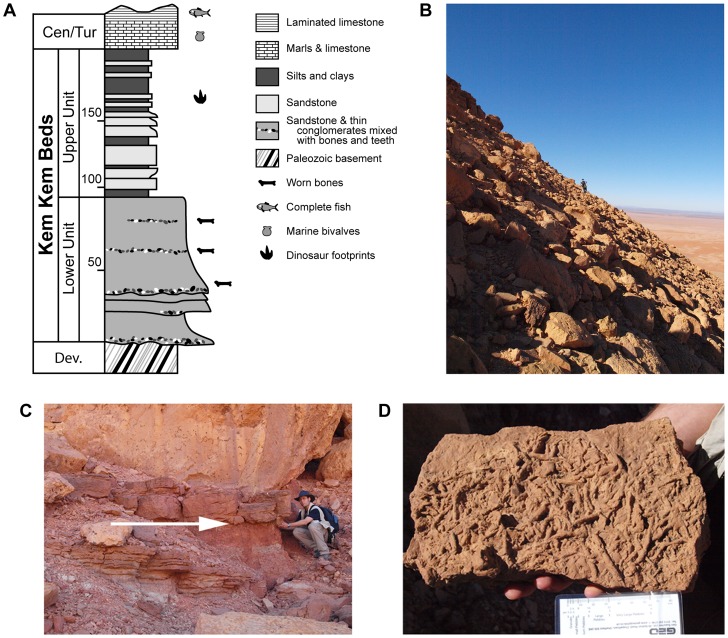
Geological context of the Kem Kem ichnofossils. A, Simplified section through the Kem Kem sequence, modified from Martill et al. (2011). B, Debris-covered slope of Gara Sbaa, at the main collecting level for dinosaur footcasts. C, Main track horizon, marked by arrow. D, Ichnofossils collected on the same horizon (burrowing structures?) Scale bar equals 8 cm in D.

The likely Cenomanian-age Upper Cretaceous ‘Kem Kem beds’ are exposed along the face of a limestone-capped escarpment extending some 250 km in length in southeastern Morocco [1], [2] (Figure 1). They comprise a 150–200 m thick sequence of fluvial siltstones and sandstones divided informally into ‘lower’ and ‘upper’ units [1], [2] (Figure 2). First extensively explored in the 1950s by French paleontologist René Lavocat, the Kem Kem beds have been subject to renewed interest and fieldwork since the mid 1990s [1], [3]. The diverse vertebrate assemblage described from the Kem Kem beds includes elasmobranchs, bony fishes (actinopterygians, coelacanths, lungfish), turtles, crocodylomorphs, pterosaurs, non-avian dinosaurs, and birds [1], [3]–[12]. The rich ichnological record preserved alongside these body fossils [1], [13], however, has not been thoroughly described. Although footprints of Jurassic and Cretaceous age have been recorded at a few other sites in Morocco [14]–[16], the Upper Cretaceous Kem Kem beds provide by far the greatest range of ichnofossil evidence that impacts recorded biological diversity and the interpretation of depositional settings (Figure 2B–D).

### Geologic Setting

The Kem Kem beds rest unconformably on Paleozoic (Cambrian through Silurian) marine strata and are capped by a cliff-forming Cenomanian-Turonian-age marine limestone [1], [2], [11], [17]–[20] (Figure 2A). The Kem Kem beds comprise a fining-upwards sequence that reflects decreasing gradient and increasing marine influence [20]. The ‘lower’ unit is dominated by coarse sandstones and occasional conglomerates. The ‘upper’ unit is finer-grained, composed of thinly-bedded sandstones, siltstones, mudstones, and rare evaporites that represent overbank, lacustrine, and tidal-flat paleoenvironments.

Although radioisotopic dating of the Kem Kem beds has not been possible, a minimum relative age of Cenomanian (ca. 95 Mya) has been inferred from the elasmobranch taxa within the beds and from the ammonite *Neolobites* in the overlying Cenomanian-Turonian limestone [1], [21].

The trace fossils described below come from several horizons in the Kem Kem beds, with the majority found in the middle portion of the upper unit at the Gara Sbaa locality [1] (Figure 1B, 2B). The ichnofossil assemblage is exposed in patchy outcrop amid the limestone debris that covers most of the scarp slope (Figure 2B).

## Materials and Methods

The ichnofossil assemblage either was collected or photographed during expeditions to southeastern Morocco in 1995 from the University of Chicago (led by PCS) and in 2008 from University College Dublin (led by NI). For permits and support of our fieldwork we thank the Ministère de l'Energie, des Mines, de l'Eau et de l'Environnement (previously Ministère de l'Energie et des Mines) and the Faculté des Sciences Aïn Chock Casablanca in Morocco. Collected specimens are curated primarily at the University of Chicago (UCRC I 173, 252–264 and 1995, temporary collection, see Table 1 for abbreviations), with one specimen in a collection at the Faculté des Sciences Aïn Chock of Casablanca (see Table 1). Footprint outlines were traced from photographs of specimens. Measurements were taken in the field and in collections (see also Figure S1). Locality data is accessioned at the Faculté des Sciences Aïn Chock (Morocco), the University of Chicago (USA) and the University of Portsmouth (UK). The individual shown on Figure 2 in this manuscript (lead author) has given written consent to appear in this publication.

**Table 1:** Measurements of tridactyl tracks from the Kem Kem beds.

UCRC I # (only rows 1–15)	ML	MW	MDe	LD II	LD III	LD IV	DII-DIII	° DII/IV
250	310	187	/	230	310	200	167	50
251	374*	275	90	300	370	280	272	48
252	225	178	80	165	225	150	162	63
253	310	161	95	185*	310	180	148*	53
254	190*	175*	110	155*	190*	135*	161*	57*
255	245	272	/	180	245	180	260	88*
256	370	255	80	255	365	250	240	54
257	335	225	178	185	335	180	190	60
258	430	301	120	240	390	240	260	57
259	285*	265*	95	185*	285*	165*	230*	79
260	377	309	60	235	345	230	250	59
261	370	250	85	220	340	215	230	58
262	318	245	80	165	240	165	225	80
263	178	187	40	130	178	130	183	77
264	291	204	65	160	260	150	160	41
FSAC-KK12	272	227	/	186	272	181	180	58
FS1	343	246	/	296	343	268	250	53
FS2	333	283	/	266	333	270	233	60
FS3	361	323	/	294	361	300	308	67
FS4	525	510	80	385	525	385	472	70

Abbreviations: De, depth, DII-IV, digits II to IV; FS, field specimen (i.e. not collected); FSAC-KK, Faculté des Sciences Aïn Chock Kem Kem collection; L, length; M, maximum; UCRC I, University of Chicago Research collection ichnofossil; W, width;° angle; * rough estimate because of substantial missing parts. Second column from the left records distance between digits II and III. Measurements in mm. Note that the poor definition and outline of the specimens mean that the values are estimates and approximate. See supporting information (Figure S1) for examples of measurements.

## Results

### Burrows, Crawling Tracks, Borings

#### Vertical Burrows (Lower Unit):

**Figure 3:**
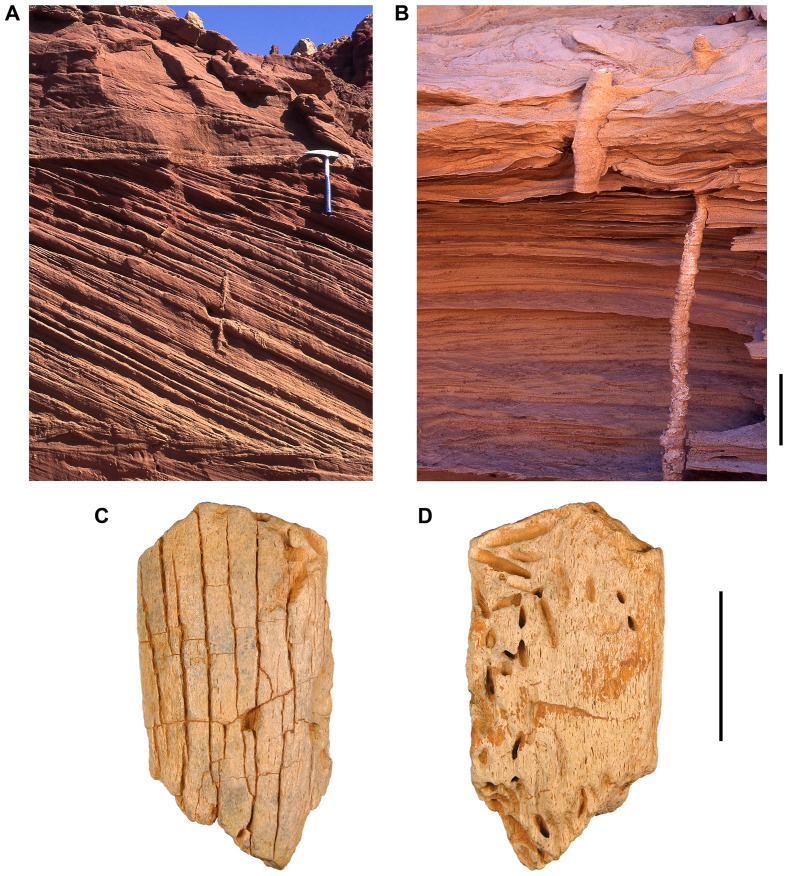
Traces of the lower Kem Kem beds. A, B, Vertical burrows within thickly-bedded cross-bedded (A) and planar-bedded (B) sandstones. C, D, Heavily bored dinosaur bone fragment. The outer cortex of this bone shows deep longitudinal cracks and a loss of the most external bone (C) indicative of stage 4 weathering of Behrensmeyer (1978) [77]. Bone fragment also shows moderate amounts of abrasion. Borings, *Cubiculum ornatus*, are most prevalent in the bone interior (D). Hammer is 33 cm long in A; scale bar equals 5 cm in B and 5 cm in C and D.

Relatively straight, cylindrical burrows with irregular cementation of their infilling and neighboring sand occur rarely in cross-stratified sandstones of the lower unit of the Kem Kem beds (Figure 3A, B). Burrow dimensions typically measure approximately 1.5 to 2 cm in diameter and over 30 cm in length. These burrows may represent aquatic dwelling traces or escape passages [22]. The lack of internal structure prevents a more definitive interpretation.

#### Bone Borings (Lower and Upper Units):

Vertebrate bone fragments are common in the lower unit and lowermost portion of the upper unit. Large bone fragments that exceed 5 cm in length frequently exhibit two postmortem features: fracturing from subaerial weathering and insect borings (Figure 3C, D). Borings usually take the form of gently arched tubes with a subcircular diameter ranging from 2.5–5.0 mm and a length up to 3 cm. Most borings enter the bone surface at a low angle. Some enter at a higher angle and form U-shaped tunnels. In all cases, the curvature of the boring is restricted to a single plane, and borings with an oval cross-section have the greater diameter in the same plane as the boring trace. When abundant, borings often crosscut one another. Several aspects of these borings argue for a subaerial, rather than aquatic, trace-maker. The borings do not exhibit the flask-shaped form that characterizes *Gastrochaenolites* and *Teredolites*, which are attributed to clams. The borings are restricted to bone from terrestrial vertebrates that show subaerial weathering and do not occur on crocodilian or fish bone. Dimensions and overall form closely match bone borings described from the Upper Cretaceous of Madagascar and assigned to *Cubiculum ornatus*, which are regarded as traces of necrophagous or osteophagous insects [23].

#### Horizontal Meniscate Burrows (Upper Unit):

**Figure 4:**
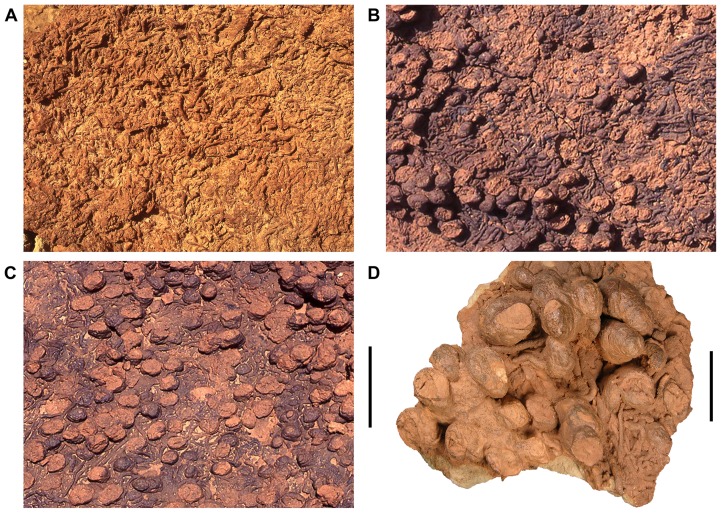
Horizontal meniscate and subvertical burrows of the upper Kem Kem beds. These burrows are preserved as convex hyporelief most commonly observed together on the undersides of weathered-out siltstone and sandstones slabs. Coverage can reach nearly 100% of the slabs. Relative abundances vary from slabs dominated by horizontal meniscate burrows (A), to more even mixes (B), and slabs dominated by subvertical burrows (C). D, Close-up of mixed assemblage showing the oval to asymmetric cross-sections of the subvertical burrows. Scale bars equal 10 cm in A–C and 4 cm in D.

Horizontal to subhorizontal, back-filled burrows are common in select horizons in the upper unit of the Kem Kem beds (Figure 4A–C). These unbranched, sinuous burrows are subcircular to elliptical in cross-section with a maximum diameter ranging from 2.0 to 6.5 mm. They lack expanded chambers and have no surface ornamentation, a distinct wall, or a regular pattern of backfill. In general, the menisci are irregular and homogenous. The meandering burrows typically have lengths between 2.0 and 7.0 cm. Rarely, these burrows occur as sparse epichnia in association with ripples and siltstone beds. Typical preservation is as hypichnia with semirelief and hyporelief on thin to medium beds of fine sandstone or siltstone overlying mud- or claystone. These beds generally erode out as float slabs on the steep weathering slopes of the Kem Kem escarpment. Abundances range from common to nearly 100% coverage of the beds base. These horizontal burrows commonly occur in varying abundances with short, blunt-ended vertical burrows (see below).

A variety of organisms including arthropods and priapulid, sipunculid, and oligochaete worms can produce back-filled burrows in marine, non-marine aquatic, and subaerial settings [24]–[30]. The absence of additional markers (distinct burrow wall, surface ornamentation, heterogenous or packeted backfill) prevents a more specific assignment to the ichnogenera *Ancorichnus*, *Scoyenia*, *Keckia*, *Laminites*, and *Naktodemasis* [24]–[27]. The horizontal burrows are most similar to the ichnotaxon *Beaconites antarcticus* from the Lower Cretaceous of England [28].

#### Subvertical Burrows (Upper Unit):

These are short, relatively straight, cylindrical burrows that taper to rounded, blunt ends in a subvertical orientation (Figure 4C). As with the vast majority of horizontal meniscate burrows, these traces occur as hypichnia on the base of fine-grained sandstone or siltstone beds that overlie mudstone deposits in the upper unit of the Kem Kem beds. Their irregular walls and occasional offsets suggest that they were made in a soft, muddy substrate, and later compacted and distorted due to sediment loading. Compaction likely greatly reduced the overall length of the burrows. Cross-sectional shape varies from subcircular to more strongly elliptical, with approximately one-third of the burrows with an asymmetrical ovoid cross-section. The long axis ranges from 14–55 mm, and the ratio of long-to-short axes averages approximately 1.5∶1 (Figure 4D). The uniform burrow fill lacks internal structure, e.g. spreiten, and suggests an open structure representing a dwelling trace. Subvertical burrows are often densely packed (690–760 burrows/m^2^) and intermingled with horizontal meniscate burrows, covering much of the surface of a bedding plane.

The subvertical burrows have an average diameter (∼40 mm) considerably greater than that typical for the vertical dwelling burrows attributed to the ichnogenus *Skolithos* [31]. They most closely resemble the burrows in marginal marine deposits attributed to decapod crabs (figure 12–2 in [32]). The steeply inclined burrows of the extant sand fiddler crab (*Uca pugilator*) are simple in form and range from 10–20 mm in diameter and 6–20 cm in depth [33]. The varying cross-sectional shape of subvertical burrows in the Kem Kem beds might reflect sexual dimorphism, given that burrow diameter differs by sex in *Uca pugilator* [33]. The asymmetric cross-section could also correspond to a bivalve, and we can not rule out this possibility, but the uniformity of dimensions through the entirety of the burrow length would argue against a bivalve origin. A decapod crab of appropriate size for these burrows has been discovered in a lakebed facies within the upper unit of the Kem Kem beds.

#### Large-Diameter Traces (Upper Unit):

**Figure 5:**
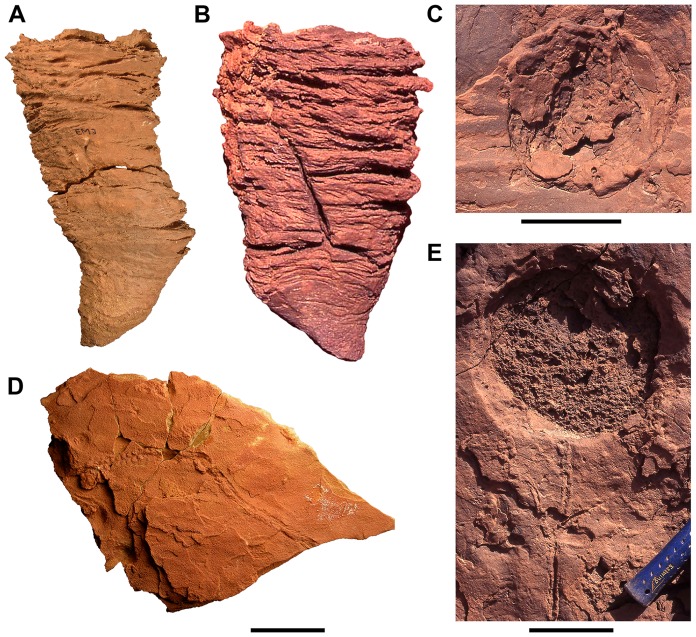
Crawling and resting traces of the upper Kem Kem beds. A–C, Lateral (A, B) and bedding plane (C) views of *Conichnus conicus* traces interpreted as sea anemone resting traces. Note the concave relief in plan view and the irregular planar to cross-bedded spreite in lateral view. Ripples and faint *Scolicia* trails are visible on the bedding surfaces in B. D, Large diameter burrow in plan view with one prominent and several less discrete *Scolicia* trails. The large burrow could represent the opening of a lungfish burrow or alternatively could represent a differently preserved *Conichnus*. E, Crawling trace leading to a vertical burrow. The alternating series of bumps of the proximal portion to burrow is reminiscent of *Scerichnites*. Scale bars equal 4 cm in A, B and D, and 9 cm in C and E.

Traces with a maximum diameter ranging from 8–15 cm occur within thin- or medium-bedded siltstones in the upper unit of the Kem Kem beds (Figure 5A–C) most abundantly about 50 km northeast of the locality Gara Sbaa near the village of Er Remlia. Several specimens as long as 18 cm have weathered out of the beds as burrow casts. Above the funnel-shaped base, the trace increases more gradually in diameter. The fill exhibits irregular, bedding-parallel to subparallel laminations with occasional ripple cross-bedding that comprise a stack of concave-upward structures.

A few large-diameter burrows are exposed on the upper surface of siltstone beds in association with ripple marks and gastropod trails (Figure 5C). Most show evidence of nested laminae. The infill of some, however, has no internal structure and may represent a distinct type of trace (Figure 5E).

The stacked laminae of increasing diameter suggest that these large-diameter traces were occupied by a relatively sessile, radially symmetrical, growing organism keeping apace with sediment aggradation (Figure 5A–C). These cubichnia are most similar to the ichnotaxon *Conichnus conicus*, which have been interpreted as the resting or dwelling traces of actinarian anthozoans, or sea anemones [34], [35]. The absence of clear medusoid symmetry distinguishes these traces from the similar *Bergaueria* and *Conostichus* [34]–[36].

Subcircular burrows on bedding surfaces that lack laminae could represent lungfish burrows [37]. Their uniform fill suggests a large sediment-filled hollow, and lungfish tooth plates are common in the lower and middle sections of the Kem Kem beds. No flask-shaped structures were observed in cross-section, however, and such structures characterize lungfish burrows [38]. So these more uniform filled large-diameter burrows may reflect a weathering variant of C*onichnus conicus*.

C*onichnus* occur in a variety of depositional environments including estuarine, fluvial- and tidal-dominated deltas, foreshore and offshore settings [34], [39]–[42]. The infilling of large-diameter burrows may provide a means of gauging sedimentation rate by comparing known growth rates of sea anemones as measured by changes in basal disk area. With this rate, one could compare the change in cross-sectional area in the Kem Kem traces versus the height (i.e. the amount of sediment accumulated during this period of growth).

#### Crawling Traces (Upper Unit):

At least two varieties of crawling traces (repichnia) occur as rare traces in the upper unit of the Kem Kem beds. The first is known from a single specimen and includes a cylindrical burrow 1 cm in diameter connected to a trail 0.8 cm wide and 14 cm long (Figure 5D). The trail shifts from convex semi hyporelief near the burrow to full hyporelief. The trail consists of two parallel rows of low, rounded, and asymmetric bumps, which are gently sloped away from the burrow and steeply sloped toward the burrow. Within each row, the bumps are spaced 3 mm apart and the parallel rows of bumps are offset so they alternate from one side to the other (see figure 2 in [43]). The portion of the trace closely resembles *Saerichnites*, which has been interpreted as a mollusk [44]. Away from the burrow, the trail gradually transitions into a series of fine striations that form a chevron pattern that diverges from a central ridge.

The second track, which occurs with greater frequency, is developed as concave epirelief on fine sandstones and siltstone beds (Figure 5E, 6A). The trail has a deep central furrow between raised edges. Several trails of this kind appear in association with large circular traces discussed below. Others are preserved on a single slab. These traces likely represent *Scolicia* sp., which is widely regarded as a crawling gastropod [30].

**Figure 6:**
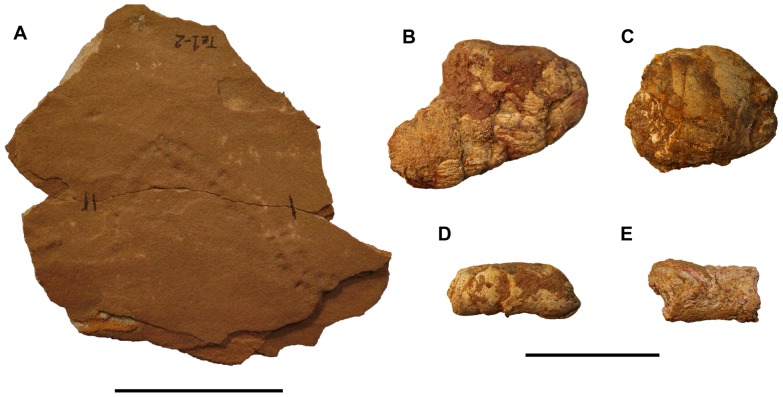
Examples of small ichnofossils from the Kem Kem sequence. A, UCRC PI714. B, coprolite FSAC-KK 935. C, FSAC-KK 936. D, FSAC-KK 2871 and E, FSAC-KK 2838. See Table 1 for institutional abbreviations. Scale bars equal 5 cm in A and 5 cm in B–E.

### Coprolites

Coprolites occur rarely in the middle of the section of the Kem Kem beds—the upper one-half of the lower unit and the lower one-half of the upper unit. They range in size from 2–5 cm to larger specimens over 10 cm in length (Figure 6B–E). Large specimens exhibit a concave end and a sinusoidal axis, which characterize the excrement of extant crocodyliforms e.g., [45], [46]. The smaller coprolites may have been generated by elasmobranchs or actinopterygians.

### Dinosaur Footprints

In 1996, Sereno et al. [1] cited the existence of a “footprint zone” approximately 80 m in thickness in the upper unit of the Kem Kem beds, mentioning in particular the presence of theropod, sauropod, and ornithopod footprints both as impressions and natural casts. In 2008, additional impressions and natural casts were discovered in the same footprint zone during an expedition led by one of us (NI). More recently, other researchers have commented on the presence and composition of Kem Kem footprints [13].

**Figure 7:**
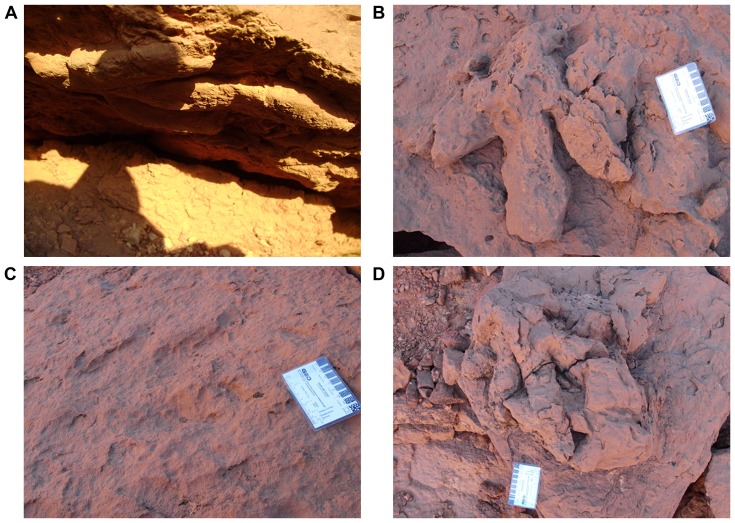
Examples of dinosaur footprints found at Gara Sbaa. A, B, Natural casts of theropod tracks (not collected). C, True track of a theropod (not collected). D, Superimposed natural casts of theropod tracks (not collected). Scale bars equal 8 cm in B–D.

**Figure 8:**
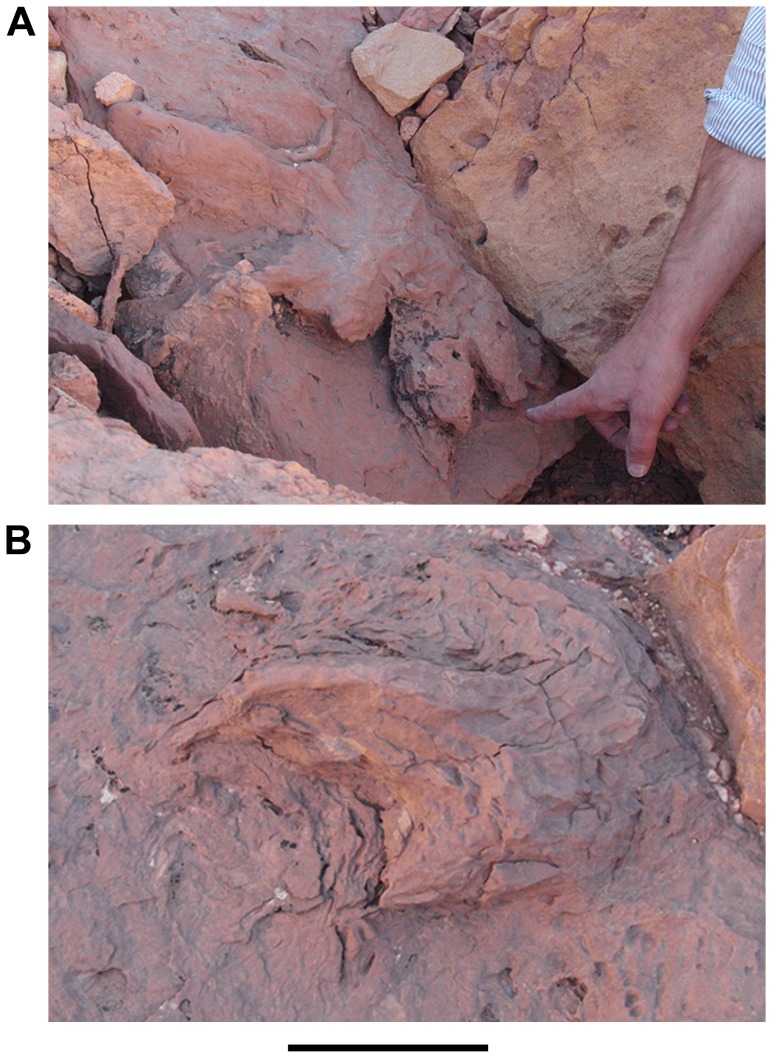
Theropod footprints at Gara Sbaa. A, Large isolated natural cast of theropod track (not collected). B, Relatively poorly defined natural cast of a theropod track (not collected). Scale bar equals 10 cm in B.

At least three track-bearing horizons occur in the upper unit of the Kem Kem beds, as best documented in a section near the village of Er Remlia, where footprints were discovered approximately 90, 60, and 10 m below the Cenomanian-Turonian limestone that overlies the Kem Kem beds (Figure 2A). The last horizon, the one closest to the limestone cap, preserves by far the greatest number of footprints. The locality Gara Sbaa (Figure 1B) preserves the greatest number of footprints, some of which have broken free at their attachment or are preserved still attached to slabs of sandstone on cliff-face talus (Figure 2B). Smaller specimens were collected, whereas others on larger slabs were documented with photographs (Figures 7, 8). Additional footprints were found at the locality Iferda Timenkherin, approximately 35 km northesast of Er Remlia, and a few footprints were found at other sites in the same zone (see also [13]).

**Figure 9:**
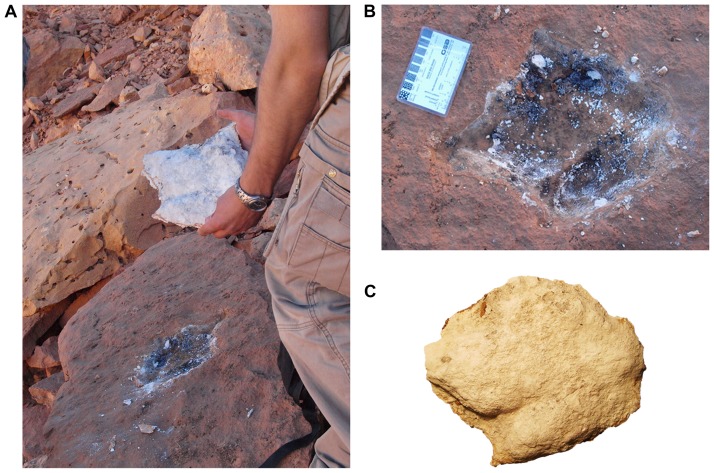
Removal of a plaster cast of a probable theropod footprint. A, Removal of a plaster cast left behind by the 1995 University of Chicago expedition. B, Appearance of the track after removal of the plaster. C, Plaster cast, now catalogued as FSAC-KK 12. Scale bar equals 8 cm in B.

**Figure 10:**
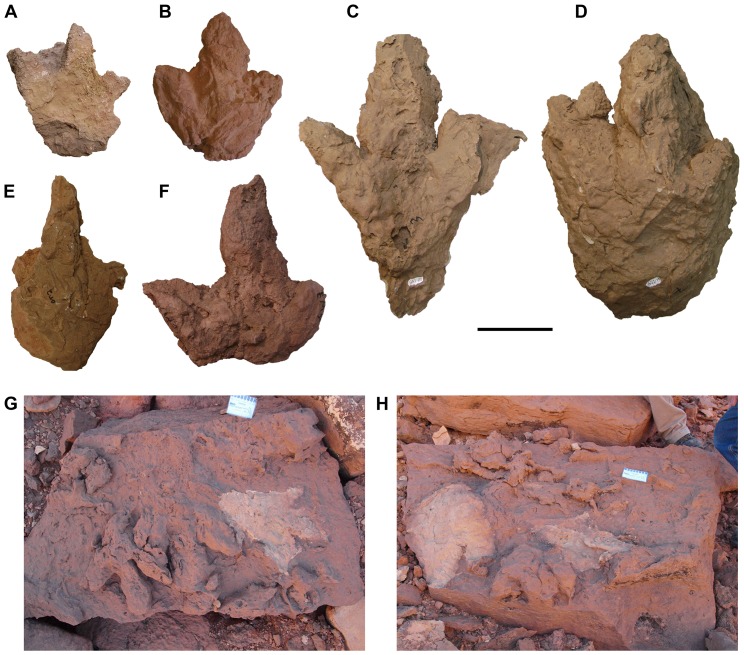
Ichnomorphological diversity of theropod tracks. A, UCRC I 160. B, UCRC I 252. C, UCRC I 260. D, UCRC I 258. E, UCRC I 253. F, UCRC I 259. G, Image of the block that contained UCRC I 260 identified during 2008 UCD/UoP/FSAC expedition. H, Image of the block that contained UCRC I 258, identified during 2008 UCD/UoP/FSAC expedition. Scale bars equal 10 cm in A-F and 8 cm in G and H.

**Figure 11:**
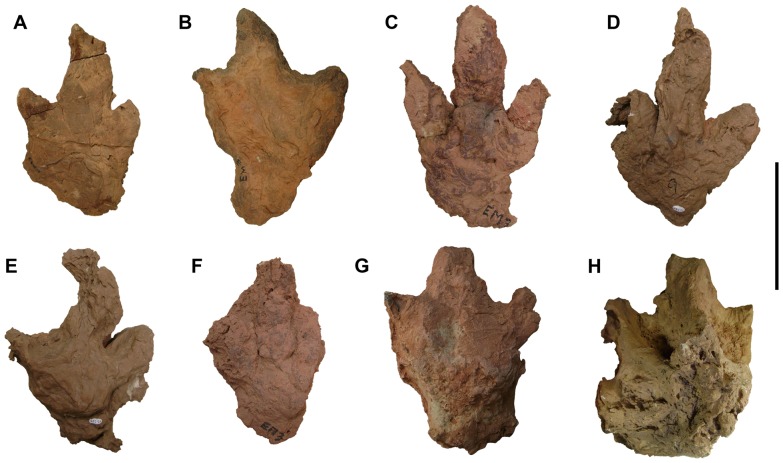
Large theropod footprints. A, UCRC I 250. B, UCRC I 251. C, UCRC I 256. D, UCRC I 261. E, UCRC I 262. F, UCRC I 264. G, H, UCRC I 255 in (G) ventral view and (H) dorsal view. Scale bar equals 20 cm.

Most of the footprints are preserved as natural sandstone casts infilling footprint impressions in an underlying soft mudstone (Figures 7, 8, 9, 10, and 11). These natural casts are hanging, or detached, from the bottom surface of the sandstone bed. As a result, there are no broad exposures of the footprint horizon; footprints are exposed *in situ* near the edge of the exposed sandstone bed and on the talus slope on overturned slabs and as single, detached footprint casts.

**Figure 12:**
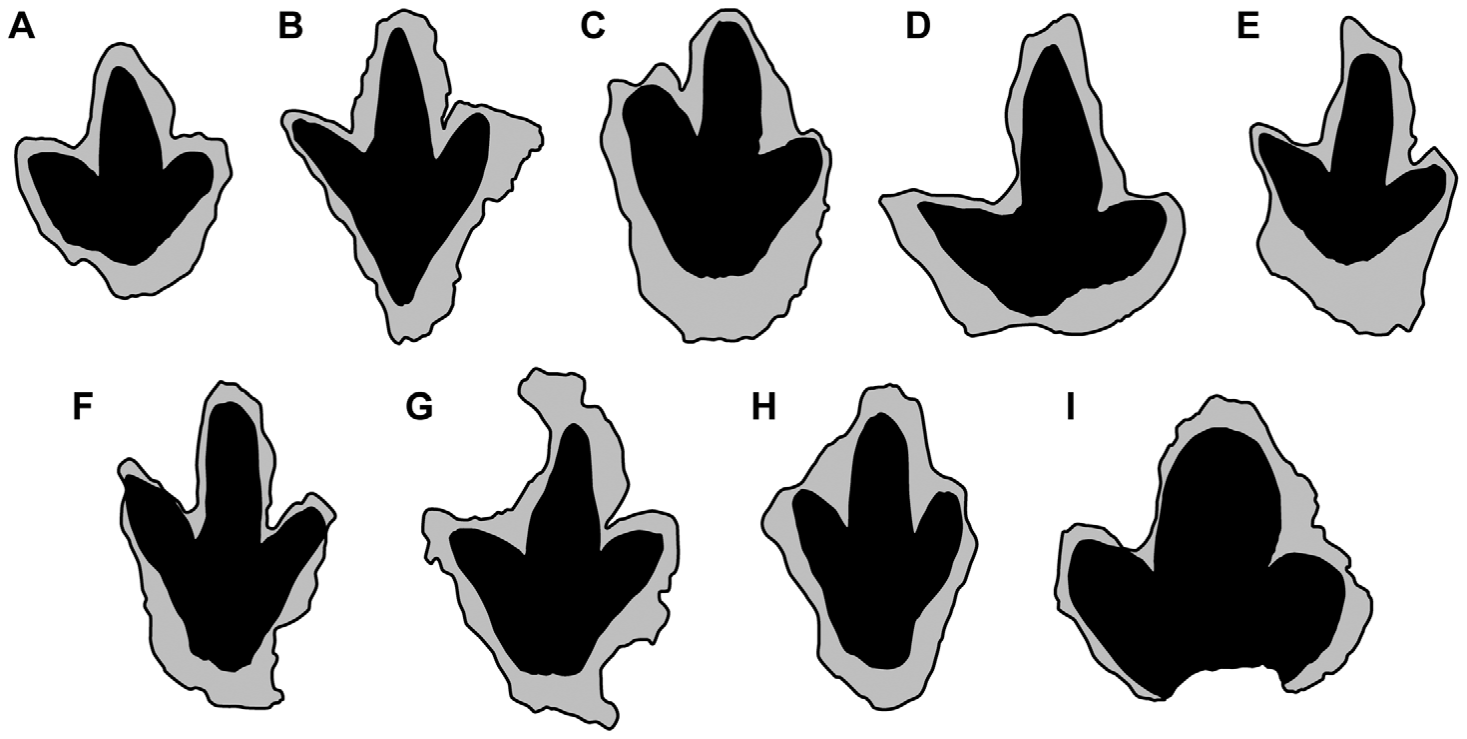
Likely digit morphology of selected Kem Kem dinosaur tracks. A, UCRC I 252. Track photo shown in Figure 10B. B, UCRC I 260. Track photo shown in Figure 10C. C, UCRC I 258. Track photo shown in Figure 10D. D, UCRC I 259. Track photo shown in Figure 10F. E, UCRC I 250. Track photo shown in Figure 11A. F, UCRC I 256. Track photo shown in Figure 11C. G, UCRC I 262. Track photo shown in Figure 11E. H, UCRC I 264. Track photo shown in Figure 11F. I, UCRC I 263. Track photo shown in Figure 16B. Preserved digit impressions in black, track outline in grey. Outlines not to scale; see Figures 10, 11 and 16 for size of specimens.

Sandstone casts that have infilled crisscrossed mudcracks suggest that the dinosaurs were walking across muddy substrates, which may have partially dried before or after passage of the trackmakers. Occasional footprint impressions were found in the sandstone layer itself, suggesting that conditions after initial deposition of sands over the mudstone were also favorable for the preservation of footprints.

#### Theropod Tracks:

The great majority of footprints collected and documented in the footprint zone of the upper unit are tridactyl and can be attributed to theropod dinosaurs on the basis of the pointed or narrow shape of the ungual impressions [1], [13]. Approximately 30 of these were too large to collect (Figure 10G, H). These tridactyl footprints are not diagnostic in any way beyond attribution to theropod trackmakers, and we do not refer them to particular ichotaxa.

The footprint impressions show considerable relief (4.0–17.8 cm), indicating that the feet of the trackmakers sank deeply into a wet substrate. A wide range of preservation is apparent. Some tracks are poorly defined, with individual digital impressions indistinct (Figure 10D), as is often the case in deeper prints [47]–[49]. In one particularly deep natural cast (Figure 11H), the movement of the pes exiting the sediment is preserved. The path of the digits and their unguals are cast in wedge-shaped relief with striations indicative of passage out of the mud. The formation of deep footprints of this sort (Figure 11G, H) has been described elsewhere [48], [49]. In some specimens, claw, toe and heel impressions are preserved (Figure 11D). The exposure of the principal track-bearing horizon is not great enough to observe trackways. On some particularly large overturned slabs of sandstone, there are many overlapping footprints of different size (Figures 7D, 10G).

**Figure 13:**
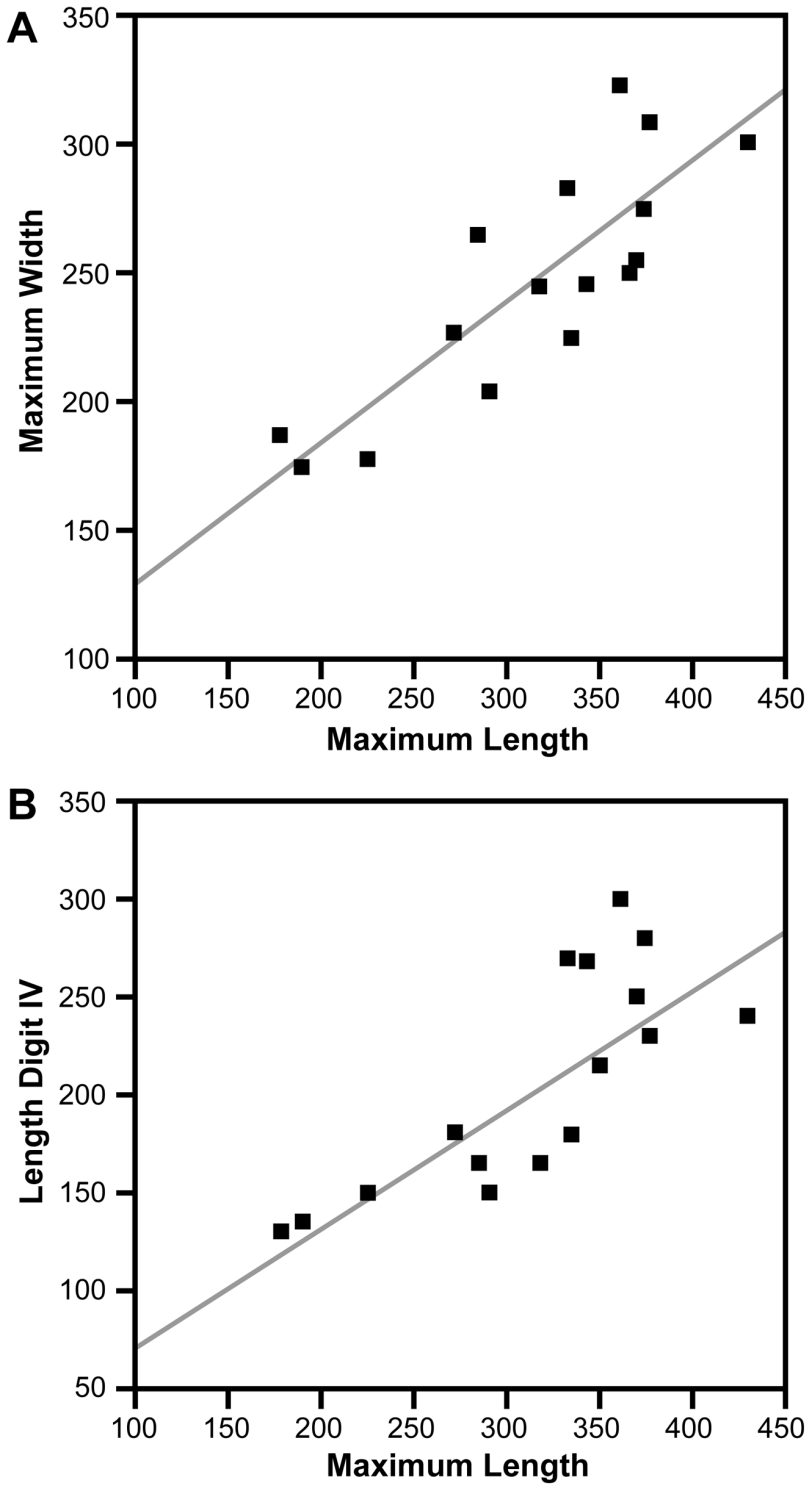
Dinosaur tracks: Width/Length and Digit IV/Maximum length graphs. Measurements in mm.

Pedal digit III is longer than digits II and IV (Figures 10, 11, 12), and total length and width range from 18–51 cm and 14–47 cm, respectively (Table 1). Some are proportionately narrow footprints with a relatively low divarication angle between pedal digits II and IV (Table 1). Width-to-length ratios are also variable, ranging from 0.67–1.05. The regular scaling between both maximum width and length of digit IV versus maximum length of the pedal print suggests that the suite of tracks may have been made by a single theropod ichnotaxon (Figure 13). A plot of maximum width against maximum length, however, shows that footprint width decreases with increasing footprint length (r = 0.84; Figure 13A). Thus smaller footprints have a width-to-length ratio of 0.92 compared to 0.74 for larger footprints. The length of pedal digits II and IV also appear to scale in a consistent manner with footprint dimension (Figure 13B). The exceptionally deep footprint cast UCRC I 257 is a notable exception, which may have been influenced by the very soft substrate.

Among Kem Kem theropods (*Carcharodontosaurus*, *Spinosaurus*, *Deltadromeus*, an abelisaurid), information on the osteology of the pes is limited to *Deltadromeus* [1]. As adults, all of the aforementioned theropods, with the possible exception of the unnamed abelisaurid, possibly referable to *Rugops*, would have generated footprints at the very largest end, and likely larger, of the size range observed in the footprint zone. The size range is approximately 2.5 times, from smallest to largest (Table 1). Most of the footprints, therefore, were generated by subadult individuals of the aforementioned theropod genera or smaller-bodied theropods that remain very poorly documented as body fossils.

#### Sauropod Tracks:

Sauropod footprints are extremely rare in the footprint zone, despite the occurrence of rebbachisaurid sauropod bone fragments among body fossils recovered in the Kem Kem beds. Two sauropod footprint casts have been identified [1] and a possible sauropod manus-pes pair of footprints was observed in cross-section northeast of Gara Sbaa near the locality Gour Lembech.

**Figure 14:**
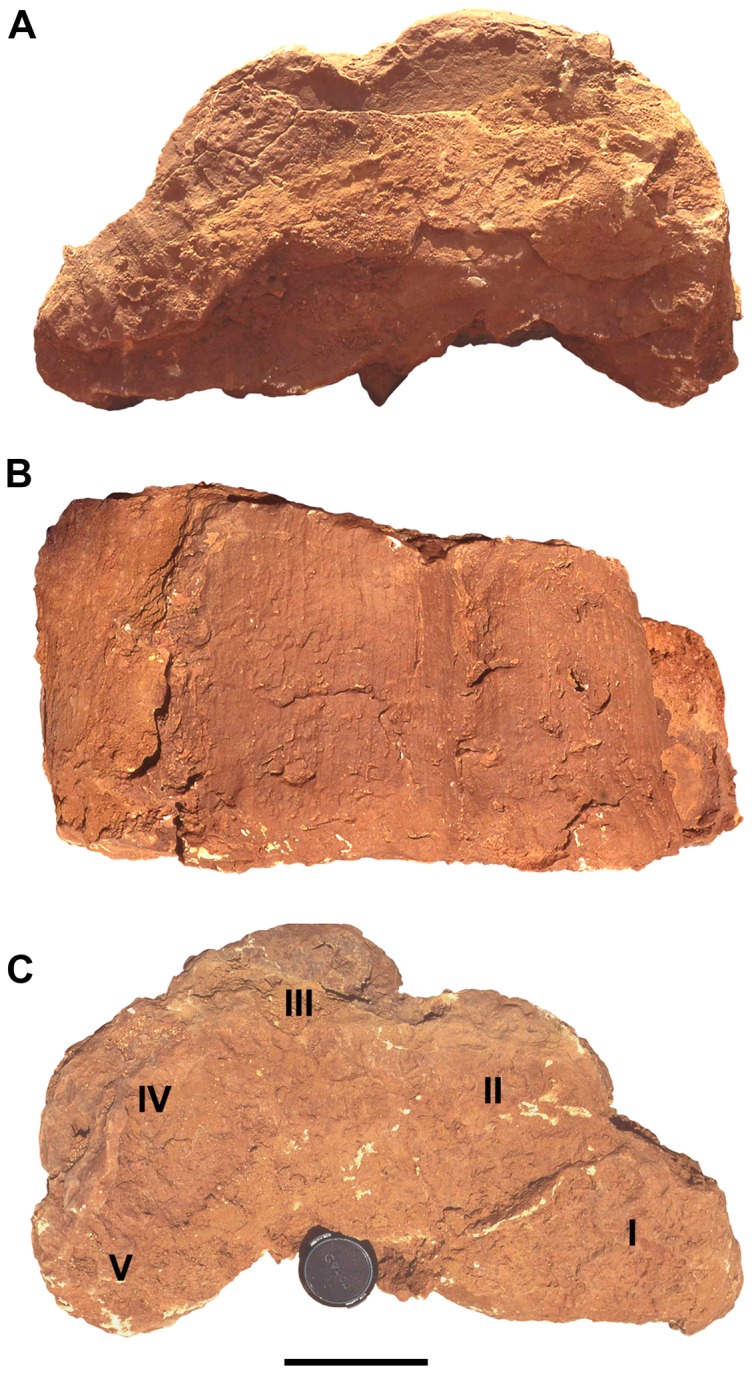
Sauropod-manus print cast (UCRC I 1995). Natural cast of sauropod left manus track in dorsal (A), anterior (B), and ventral views (C). Note the straight grooved sides of the track in anterior view (B), the five lobes corresponding to the tightly bound digits I–V, and the absence of any divergent phalanges or unguals. Scale bar equals 10 cm.

A right manual footprint cast (UCRC I 1995) that is 23 cm in depth was found about 8 km northeast of Gara Sbaa, measuring 53 cm in width and 31 cm in length (Figure 14). The footprint has nearly vertical sides marked by shallow grooves or sulci with a subtle texture of low vertical ridges. (Figure 14B). The ridges probably were created when the manus was pulled out of the footprint impression. Five distinct lobes are best seen in ventral view of the footprint cast (Figure 14C). They represent the distal ends of metacarpals 1–5 generated by the near vertical metacarpus of a neosauropod. On the medial side of the manus footprint cast, the portion attributable to metacarpal 1 has a subtriangular shape and projects more prominently than the others. The casts left by the ends of metacarpals 2, 3, and 4 are broadly convex and subequal in width (∼17 cm). The cast for the end of metacarpal 5 is approximately the same width but has a more strongly arched perimeter with its central axis projecting posterolaterally (Figure 14C).

Although this Kem Kem track shares a crescentic outline somewhat similar to those of *Tetrapodosaurus* and *Stegopodus*, footprints typically associated with armored dinosaurs [50]–[52], the Moroccan track differs in two key features. The Kem Kem track is very deep with vertical sides, and its toe impressions are reduced to digital pads that project only minimally from the body of the track. Instead, this track suggests an animal lacking or with greatly reduced digits, and thus is unlikely to represent a thyreophoran. The crescentic form, shorter than wide proportions and inwardly directed digit I is more consistant with the manus impression of *Deltapodus* [53], and suggests that it was made by a neosauropod with a transversely arched, digitigrade manus [54]. The absence of any indication of unguals on digits I–III or of phalanges on any of the digits suggests that the trackmaker was possibly a titanosaurian sauropod [55], but we note that the apparent complete absence of unguals or phalanges is not uncommon in sauropod trackways. The Kem Kem print is proportionately broader and less arched than many neosauropod manual footprints [54], although to which degree manual footprints are arched has not been regarded as a reliable taxonomic indicator [55].

**Figure 15:**
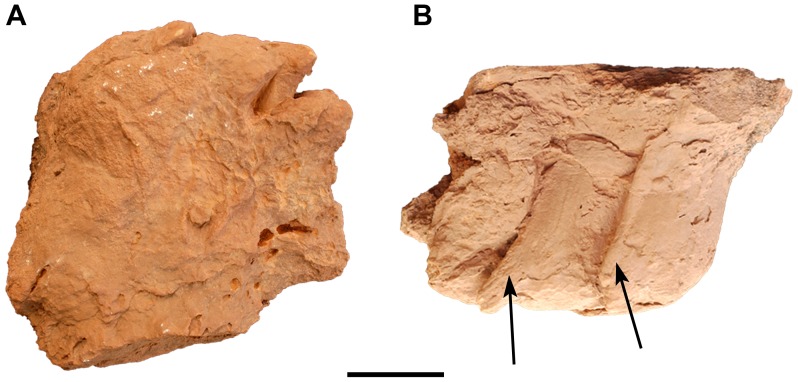
Isolated neosauropod track (UCRC I 173). A, Vental view. B, Anterior view, arrows showing claw marks. Scale bar equals 10

The natural cast of a sauropod pedal footprint (UCRC I 173) measures 81 cm and 68 cm for major and minor axes (Figure 15). There are distinct claw impressions on the inner three digits (Figure 15A) that appear to be angled anterolaterally (Figure 15B). The pedal footprint cast is not as diagnostic as the manual footprint cast and could have been generated by a diversity of neosauropods [54], [55].

#### Ornithopod Tracks:

**Figure 16:**
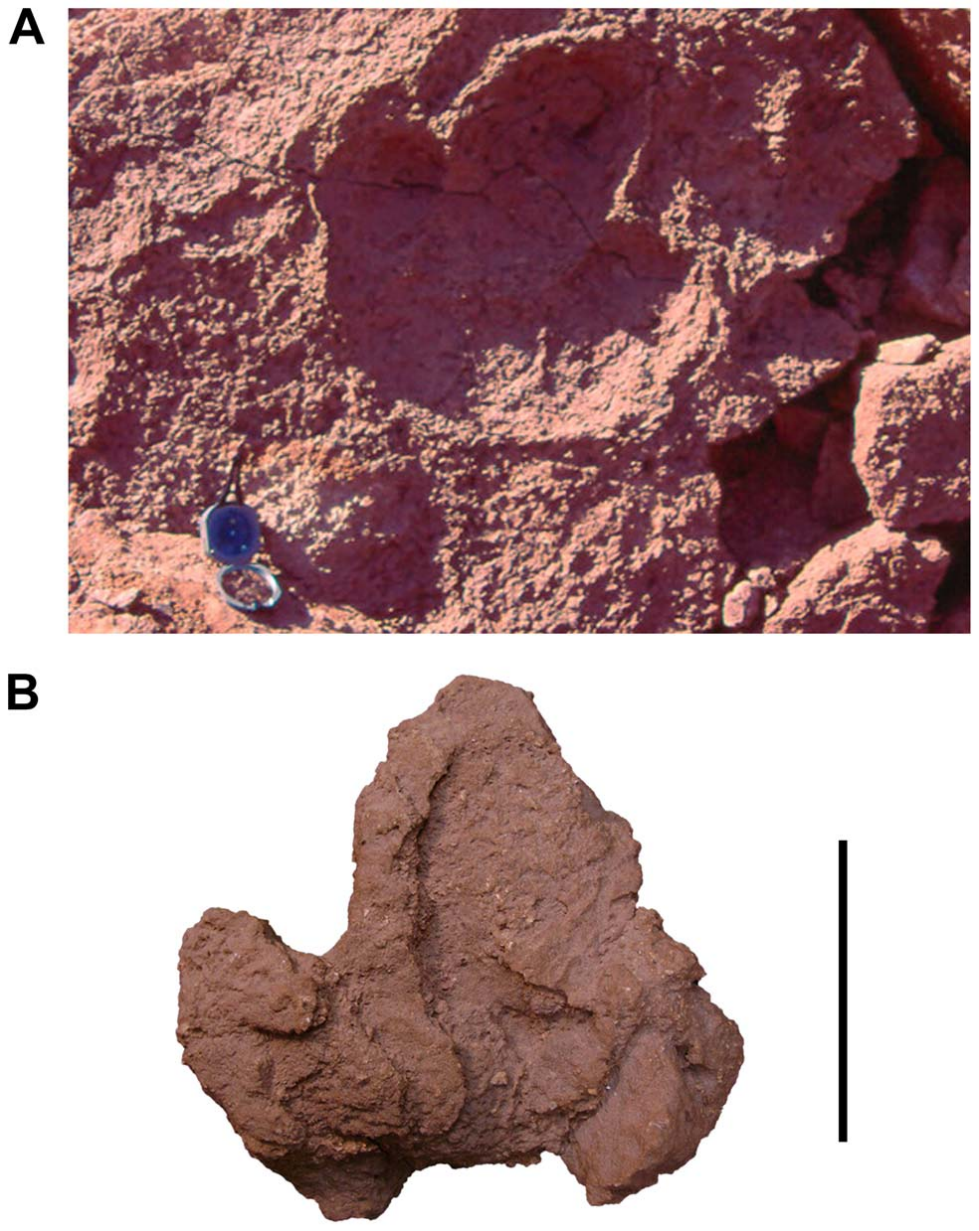
Ornithopod tracks. A, Definitive ornithopod track (not collected). B, Possible ornithopod track (UCRC I 263). Measurements of the track in (A) are provided in the text. Scale bar equals 10 cm in B.

Ornithopod pes tracks are rare in the Kem Kem. Thus far, only one definitive, and one possible ornithopod print have been found (FS4, see Table 1, Figure 16). These tracks can be recognized as ornithopods by their three divergent and subequal digit impressions that form cloverleaf–shaped outline [1], [56]. The larger, definitive ornithopod track specimen (Figure 16A) was discovered in 1995. It is noteworthy for its clear outline and considerable size (ca 50×50 cm), in which the digits are rounded terminally and relatively short (Table 1). The maximum depth of the impression is approximately 8.0 cm. A second track, UCRC I 263, was possibly made by an ornithopod trackmaker. Although there are some similarities to theropod tracks, the rounded edges of the digits and the proportionally broad and large digit imprints favor an interpretation as a small ornithopod (Figure 16B). It would represent a much smaller individual than the one described above. In size and shape the large Kem Kem ornithopod track is comparable to those typically attributed to *Iguanodon* [56], suggesting that an ornithopod of similar size frequented the Kem Kem area.

Belvedere et al. ([13]: 54, fig. 2D–F) recently reported three tracks from Gara Sbaa they regarded as made by ornithopods, identifying them based on their broader interdigital angle and thus proportionately broader track width. By themselves, these are not reliable features to distinguish ornithopod from theropod trackmakers. In the single track they figured, pedal digit III has a clearly marked, narrow, deep impression made by a trenchant pedal claw. The claw impression is clearly visible and this feature does not appear to have been caused by susbtrate deformation such as sediment collapse around the edges of digit impressions. This track cannot be attributed with any confidence to an ornithopod trackmaker.

## Discussion

### Footprint Zone

The Kem Kem beds record the transition from full subaerial exposure and erosion of the underlying Paleozoic rocks to fully marine conditions in the overlying echinoid-rich limestone. Traces are uncommon within the coarse, sandstone-dominated lower unit. Besides rare vertical burrows within cross-bedded sandstones, the most abundant traces are those of osteophagous invertebrates on subaerially exposed bones deposited within channel lags.

The upper Kem Kem unit exhibits an overall fining upward sequence likely reflecting a combination of lowered overall gradient, decrease in clastic input, and a rise in eustatic sea level. Marine influence is increasingly represented up section by the presence of mud-drapes, “flaser bedding”, and inclined heterolithic bedding. The suite of traces reflects the decreasing energy of the system as well as the increasing marine influence. The invertebrate traces of the upper Kem Kem include *Conichnus*, a suggested sea-anemone resting trace; *Scolicia*, gastropod trails; horizontal meniscate burrows possibly representing *Beaconites antarcticus*; and short, sub-vertical burrows, dwelling traces of possibly fiddler-crab or similar organisms. These last two burrows often occur together in extreme abundances representing nearly 100% coverage. Respectively, these two traces likely represent feeding and dwelling traces of tidal flat detritivores. Their occurrence high in the formation likely reflects a shift from a fluvial system to a low energy coastal setting. The persistence of dinosaurs as represented by common tracks within the upper Kem Kem reflects the transitional aspect of the sequence.

The prevalence of ichnofossils in the upper unit of the Kem Kem beds may be a consequence of its finer-grained, more heterogeneous sedimentology (interbedded mudstones, siltstones, fine sandstones) that may have facilitated preservation. The footprint horizons, which vary from one to three in sections of the upper unit and are not laterally continuous for kilometers, represent local conditions conducive to preservation of traces fossils.

### Vertebrate Trackmakers

Tracks other than those pertaining to non-avian dinosaurs are rare. We question the recent identification of pterosaur and crocodyliform tracks. Belvedere et al. reported “probable pterosaur” pedal tracks with “up to four short digit impressions” ([13]: 56, fig. 2M–O). Clear digital impressions, however, are not apparent, nor is there any indication in these impressions of the diagnostic manual track and sprawling trackway that characterize pterosaurs. Belvedere et al. identified another “partial print” as pertaining to a crocodyliform ([13]: 56, fig. 2J–L) by its sharp claw impressions and the fact that digit III is the longest of the digital impressions. These track features, however, are not diagnostic for crocodyliforms, although a crocodyliform trackmaker cannot be ruled out. A great diversity of crocodyliforms are known from the Kem Kem beds, including large aquatic species and more terrestrial notosuchians. Belvedere et al. argued, furthermore, that the track may have been made by a crocodyliform similar to *Laganosuchus* or *Sarcosuchus*, which they stated to be “well known from the Kem Kem beds” ([13]: 56). The specialized flat-skulled genus *Laganosuchus*, however, is known only from single or fragmentary portions of the lower jaw in the Kem Kem beds and in likely Cenomanian-age beds in Niger [57]. Likewise, diagnostic remains of the large-bodied genus *Sarcosuchus* are lacking in the Kem Kem beds; current evidence for this genus in Africa is limited to Aptian-Albian age beds in Niger [7]. The identification of the vertebrate trackmaker of this partial print in our opinion remains entirely uncertain.

Belvedere et al. reported turtle tracks from the upper unit ([13]: 55, fig. 2G–I). Turtle tracks are rare in the fossil record, and these resemble turtle tracks created in the laboratory [58]. Belvedere et al. reported that they were the second most common track type in the Kem Kem. In our extensive survey of the footprint zone, however, we have yet to encounter a single turtle track. They most likely occur rarely or in isolated areas of the Kem Kem footprint zone. Belvedere et al. suggested that the Kem Kem tracks resemble the turtle ichnogenus *Emydhipus* from the Late Cretacoeus of Spain, [58], although they did not cite any identifying features. The Kem Kem tracks are particularly large, in some cases exceeding 10 cm in length and width. These dimensions are nearly twice those of the Spanish turtle tracks [58]. Several subaquatic turtles, which often have sharp claws, are known from body fossils in the Kem Kem. None of the known chelonian body fossils could reasonably be expected to have left manual or pedal tracks 10 cm in length [11]. This chelonian trackmaker may not be represented among known body fossils.

The single large ornithopod footprint (FS4, Figure 14A) most likely was made by a taxon close in size to *Iguanodon* [56], [59] with a body length of approximately 9–11 meters. To date no definitive ornithopod body fossils have been reported from the Kem Kem beds. Belvedere et al. [13] incorrectly stated that ornithopod body fossils were cited by Sererno et al. [1].

Most of the Kem Kem tracks are small- to medium-sized theropod tracks (see Table 1) with a body length of approximately 4–6 m (Figures 7, 8, 9, 10, 11, 12). Small- to medium-sized theropod taxa known from body fossils in the Kem Kem beds include dromaeosaurids and abelisaurids [60]–[62], the former a tentative identification based only on isolated teeth. As dromaeosaurids are functionally didactyl, there are no tracks in the Kem Kem assemblage that can be referred to this family [63]. The pedal anatomy of the Kem Kem theropod *Deltadromeus* is known but would not generate a tridactyl track distinguishable from many other theropods. Because of substantial variability introduced by substrate material properties, pedal movement and speed, and individual variation, we prefer not to identify or make specific reference to ichnogenera here. Belvedere et al. ([13]: 55) suggested some tracks might be referable to “cf. *Megalosauripus*”, an ichnogenus based on Late Jurassic footprints from Germany. No specific features were identified for this referral, which we regard as questionable. Large three-toed tracks consistent with the estimated adult body size of the Kem Kem genera *Carcharodontosaurus*, *Deltadromeus*, or *Spinosaurus* are conspicuously absent [1]. Such tracks would likely equal those attributed to *Tyrannosaurus* [64].

### Kem Kem Track Record

Belvedere et al. ([13]: 52) suggested a “novel ichnological approach” that accepts the taxa recorded in a footprint horizon as “a more precise paleoecological sample”. Not only is there more than a single footprint horizon in the Kem Kem beds and, therefore, more than a near-instantaneous ichnological record, discernible trackmakers are relatively few and could not possibly provide an accurate representation of vertebrate faunal diversity. Read literally, we might presume that for a substantial period of time the Kem Kem fauna was dominated by a few small theropod dinosaurs and a very large turtle taxon.

The trace fossil record, to the contrary, provides an independent source of information on the presence and abundance of Kem Kem vertebrates from that obtained from body fossils. It consists almost exclusively of dinosaur tracks that are dominated by small- to medium-sized theropods [1]. Track records, however, do not necessarily accurately reflect faunal abundances, even in better-sampled ichnological assemblages [65], [66].

### Abundance of Different Dinosaur Tracks

Although the majority of Cretaceous track sites are pre-Cenomanian in age [67]–[69], Cenomanian tracksites have been reported from Brazil [70], with theropod tracks being the most abundant. In Argentina [71], Albian-Cenomanian sauropod tracksites have been reported. In the United States, Lee [72] reported on Cenomanian avian and non-avian dinosaur (hadrosaur and theropod) tracks. The Cedar Mountain Formation in the United States has also yielded diverse Lower Cretaceous trackways [73], including theropod, sauropod, ornithopod, ankylosaur and dromaeosaurid tracks. A Cenomanian theropod and ornithopod tracksite has been reported from the Yukon in Canada [74], and Cenomanian tracks have also been reported from Croatia and Italy [75], [76], where the track assemblages are dominated by sauropods, which represent about three quarters of the total tracks [77].

The Kem Kem, which so far consists almost exclusively of theropod tracks, appears to be less diverse than some other assemblages and most similar to the theropod-dominated track assemblages from the Cenomanian of Brazil (see above). The small sample size and lack of associated tracks in the Kem Kem precludes meaningful comparisons; other than that the sequence appears to be largely dominated by theropod footprints, a trend also observed in the body fossil record ([1], [13], [78], [79], contra [80]). Lockley [68] noted that in Lower Cretaceous sites in Brazil, Bolivia and Texas, theropod tracks also greatly outnumber those of herbivorous dinosaurs (sauropod, and ornithischians) by a factor ranging from approximately 2∶1 (in Texas) to 7∶1 (Bolivia). The ichnoassemblage of the Kem Kem dinosaurs parallels these assemblages with a similar prepoderance of theropod tracks [68], [74], but the sample is too small to allow robust comparisons. Lockley [68] suggested that ornithopod tracks are more abundant in European sites, whereas theropods tracks are more abundant in South American sites. Souza Carvalho [70] suggested that theropods may preferentially inhabit or visit low floodplain areas to account for the abundance of Cenomanian tracksites in Brazil. In the Jurassic age Iouaridène Formation in Morocco, theropod tracks also dominate. Among approximately 800 tracks, stegosaurus and sauropods account for only 2 and 200 tracks respectively [81]. These ichnite abundances may not reflect the relative abundance of theropods, sauropods, and ornithischians in a given ecosystem, but rather subtleties in behavior that allow theropods to contribute more than other dinosaur groups to the ichnological record.

The re-occurrence of this pattern in geographically and temporally widespread localities suggests potentially an underlying biotic or taphonomic origin. In the case of the Kem Kem ichnological assemblage, the preponderance of theropods may correspond to the shift to a marginal marine environment that favors the presence of scavengers and other carnivores over large, terrestrial herbivores. Factors may include theropod foraging behavior, the scarcity of suitable vegetation for herbivores, and the absence of suitably hard substrates for large herbivores.

## Supporting Information

Figure S1 **Principal distances measured, shown on two selected specimens.** Arrows indicate uncertain end point. Perpendicular lines indicate well-defined margin.
(TIF)
