# Correction: Dinosaur Footprints and Other Ichnofauna from the Cretaceous Kem Kem Beds of Morocco

**DOI:** 10.1371/journal.pone.0104288

**Published:** 2014-07-28

**Authors:** The PLOS ONE Staff

The name of the fourth author should read Jeffery A. Wilson.

The eighth sentence of the second paragraph of the sub section “Abundance of Different Dinosaur Tracks” should read: “Among approximately 800 tracks, stegosaurs and sauropods account for only 2 and 200 tracks respectively [81].”

The acknowledgements section should include the following: We are grateful to Andrew Milner, Lisa Buckely and Andrew Farke for comments and suggestions that improved earlier versions of the manuscript. No competing interests declared.
