# Supplementary figures and images for: Dinosaur Footprints and Other Ichnofauna from the Cretaceous Kem Kem Beds of Morocco
Source: PLoS One. 2014 Mar 6;9(3):e90751. doi: 10.1371/journal.pone.0090751 (PMC3946209; doi:10.1371/journal.pone.0090751)

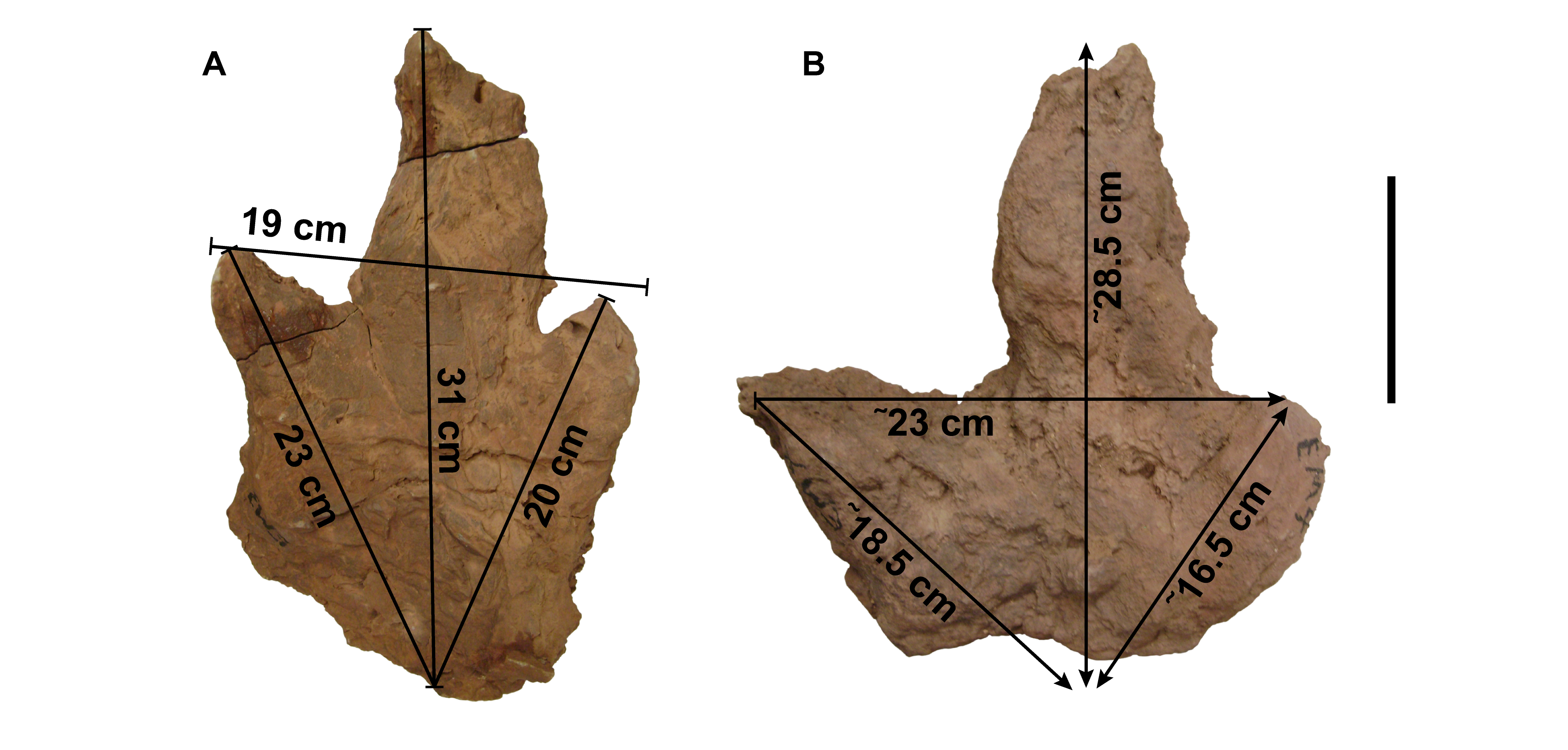

Supplement: Figure S1 — Principal distances measured, shown on two selected specimens. Arrows indicate uncertain end point. Perpendicular lines indicate well-defined margin. (TIF) [file pone.0090751.s001.tif]
